# A pilot study on adjunctive use of parametric colour-coded digital subtraction angiography in endovascular interventions of haemodialysis access

**DOI:** 10.1186/s12880-018-0270-8

**Published:** 2018-09-15

**Authors:** Ru Yu Tan, Tze Tec Chong, Fu Chieh Tsai, Suh Chien Pang, Kian Guan Lee, Apoorva Gogna, Alicia Huiying Ong, Chieh Suai Tan

**Affiliations:** 1 Department of Renal Medicine, Singapore General Hospital, Singapore, Singapore; 2 Department of Vascular Surgery, Singapore General Hospital, Singapore, Singapore; 3 Department of Vascular and Interventional Radiology, Singapore General Hospital, Singapore, Singapore; 4 Duke-NUS Medical School, Singapore, Singapore

**Keywords:** Colour-coded, Digital subtraction angiography, Haemodialysis, Vascular access, Endovascular intervention

## Abstract

**Background:**

Two-dimensional digital subtraction angiography (DSA) is the gold standard for angiographic evaluation of dysfunctional haemodialysis access. We aim to investigate the utility of parametric colour coded DSA in providing hemodynamic analysis during haemodialysis access interventions.

**Methods:**

We retrospectively studied 20 patients who underwent access intervention and applied parametric colour-coding on selected DSA acquisitions before and after percutaneous transluminal angioplasty (PTA). The difference in time to peak (dTTP) contrast enhancement and time attenuation curve (TAC) of pre- and post-stenotic regions of interest (ROIs) were obtained and compared after treatment.

**Results:**

Improvements were seen in mean percent of stenosis after PTA (*p* < 0.0001) for all cases. Median dTTP improved from 0.52 (IQR 0.26, 0.8) to 0.25 (IQR 0, 0.26) seconds (*p* = 0.001). Median 50% contrast washout time improved from 0.77 (IQR 0.39, 1.17) to 0.42 (IQR 0.23, 0.59) seconds (*p* = 0.031). Significant correlation was seen for dTTP vs. percent of stenosis (*r* = 0.723, *p* = 0.043) pre-PTA and for change in dTTP vs. percent change in stenosis post-PTA (*r* = 0.786, *p* = 0.021) for inflow lesions. Such correlation was however not seen in outflow lesions.

**Conclusions:**

Adjunctive use of parametric colour-coded DSA may provide potentially useful hemodynamic information during vascular access interventions. Larger prospective studies are needed to validate our findings.

## Background

Vascular access dysfunction remains a major contributor of morbidity and hospitalization in end stage renal failure (ESRF) patients on hemodialysis [1]. Stenosis secondary to neointimal hyperplasia can occur within the dialysis access, resulting in stenosis and thrombosis [2, 3]. Percutaneous transluminal angioplasty (PTA) of stenosis is the current standard of care as it is effective and less invasive when compared to open surgical techniques [4]. In carefully selected patients, some of these interventions can be performed in an outpatient setting [5]. Two-dimensional digital subtraction angiography (DSA) is the gold standard imaging modality during percutaneous assessment and intervention of vascular access [6]. Propagation of contrast agent through vessels or grafts during angiographic sequences is used to assess the patency of the vascular access visually, followed by determination of severity of stenosis and percutaneous intervention if required.

Anatomic measurement of luminal narrowing has been widely used to determine the severity of vascular access stenosis. Any stenosis causing greater than 50% luminal reduction should be treated [4]. Anatomic reduction of vessel diameter however may not be hemodynamically or clinically significant. Overzealous treatment of inflow stenosis especially on upper arm access may precipitate steal syndrome and increase risk of rupture [7]. Conversely, inadequate dilatation of stenosis may result in early or repeated recurrence of stenosis. Combination of anatomic with hemodynamic or clinical criteria is therefore recommended for assessment of disease severity during dialysis access interventions [8].

Using enhanced technology, DSA sequences can be post-processed to enable hemodynamic assessment of vasculature patency to aid planning of intervention during PTA. Parametric colour coding can be applied using computer software to convert conventional DSA sequences into colour images and automate quantitative hemodynamic analysis. The temporal evolution of the contrast agent at a fixed position can be recorded in a pixel-specific time-intensity curve, computed mathematically and visualized as a parametric image. The flow of contrast captured by DSA in multiple sequences will be combined into one single colour coded image which is used for hemodynamic quantification.

Parametric colour coding of DSA has been successfully used in angiographic assessment and treatment of neuroendovascular procedures and peripheral arterial disease [9–12]. However, limited information is available on adjunctive use of parametric colour coded DSA during percutaneous treatment of hemodialysis vascular access. We hypothesize that parametric colour coded DSA enables quantification of hemodynamic changes during PTA of hemodialysis vascular access. This pilot study was performed to examine the feasibility of using the difference in time to peak (dTTP) contrast enhancement and time attenuation curve (TAC) derived from colour-coded DSA to assess hemodynamic changes of stenosis in relation to anatomical changes before and after angioplasty.

## Methods

### Case selection, procedures, DSA acquisitions and post-processing

This is a single center retrospective study involving interventions performed for dysfunctional hemodialysis accesses. DSAs during the procedures were acquired as per standard hospital protocol for hemodialysis access interventions. In general, procedures were performed under local anaesthesia with aseptic preparation using a single plane angiography system (Artis one, Healthcare Sector, Siemens AG, Forchheim Germany). Prior to the intervention, bedside ultrasound was performed to determine the site of the lesions. For patients with only inflow stenosis, a vascular sheath was placed in the peripheral outflow vein in a retrograde direction and a catheter and a guidewire were advanced into the feeding artery. DSA was acquired with contrast injection via the catheter situated in the feeding artery to document the severity of the lesion. Following treatment with balloon angioplasty, post-intervention DSA was acquired with the catheter at a similar position to document the results. For patients with outflow stenosis, a vascular sheath was placed in an antegrade direction and DSA was acquired with contrast injection through the sheath. A catheter and a guidewire were then advanced centrally via the sheath. Following intervention with balloon angioplasty, post-intervention DSA was acquired with contrast injection through the same sheath. For patients with both inflow and outflow lesions, the outflow lesions were treated prior to inflow lesions. All DSA acquisitions were done with hand-injection of contrast. The angiographic data and images of each patient were stored in a dedicated work station within the centre, running software for both standard angiography and parametric colour-coded DSA.

Cases were selected by the following criteria: the procedure must be performed by the same operator throughout to exclude inter-operator variability during contrast injection, DSA sequences on intervened segment must have identical projection, and magnification and similar catheter positions in the pre- and post-PTA studies. Contrast injection must also be with catheter in proximity to the stenotic lesion. Given that the presence of multiple stenoses may interfere with interpretation of hemodynamic parameters, only DSA sequences of the last treated lesions were used for hemodynamic assessment. Procedures complicated by rupture of vessels and deployment of vascular stents were excluded in the study.

Of the 20 patients studied, 15 patients have arteriovenous fistula (AVF) and 5 have arteriovenous graft (AVG). The baseline demographics and characteristics of the patients were obtained from electronic medical records.

### Anatomic evaluation using conventional DSA acquisition (Fig. 1)

Anatomic measure of disease severity and treatment success was determined using the measurement and magnification tools on the workstation.

**Fig. 1:**
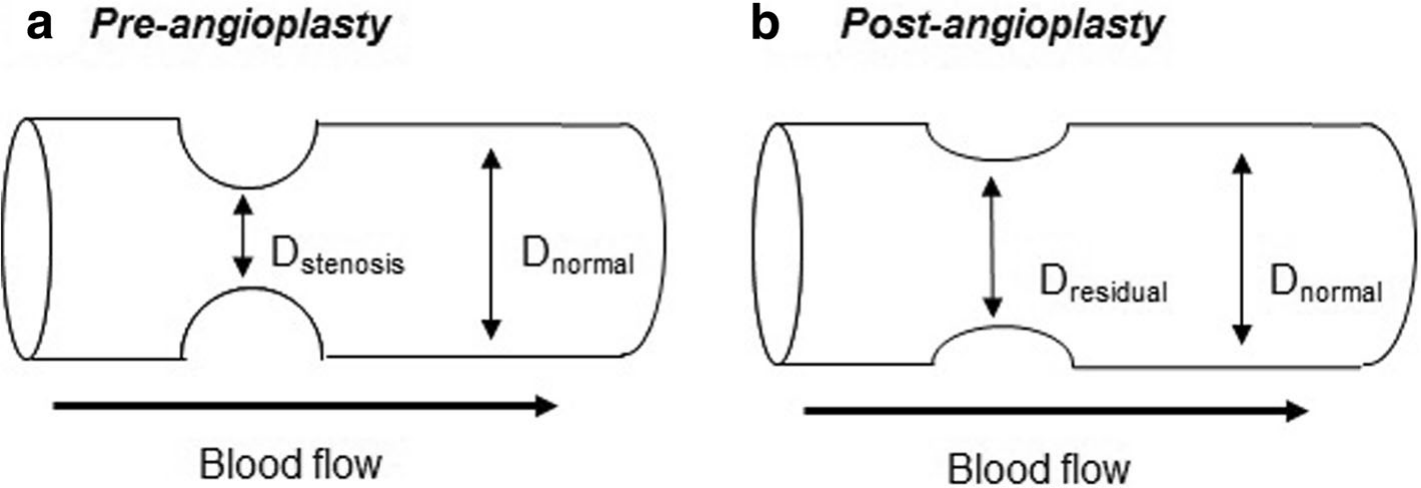
Anatomic measure of disease severity and residual stenosis. (**a**): Diameter of stenotic and normal segment were measured to estimate the percent of stenosis pre-angioplasty. (**b**): Diameter of residual stenosis and normal segment were measured to estimate the percent of stenosis post-angioplasty

#### Pre-treatment evaluation

Diameters of the most stenotic (D_stenosis_) and normal (D_normal_) segments of the vessels/grafts were obtained

$$ \mathrm{Percent}\ {\mathrm{of}\ \mathrm{Stenosis}}_{\mathrm{pre}-\mathrm{angioplasty}}=\left[1-\left({\mathrm{D}}_{\mathrm{stenosis}}/{\mathrm{D}}_{\mathrm{normal}}\right)\right]\times 100 $$

#### Post-treatment evaluation

Diameters of the residual stenosis (D_residual_) and normal (D_normal_) segments of the vessels/grafts were obtained

$$ \mathrm{Percent}\ {\mathrm{of}\ \mathrm{Stenosis}}_{\mathrm{post}-\mathrm{angioplasty}}=\left[1-\left({\mathrm{D}}_{\mathrm{residual}}/{\mathrm{D}}_{\mathrm{normal}}\right)\right]\times 100 $$

#### Evaluation of treatment success

Change in Percent of Stenosis:

$$ \left[\left(\mathrm{Percent}\ {\mathrm{of}\ \mathrm{Stenosis}}_{\mathrm{pre}-\mathrm{angioplasty}}-\mathrm{Percent}\ {\mathrm{of}\ \mathrm{Stenosis}}_{\mathrm{post}-\mathrm{angioplasty}}\right)/\mathrm{Percent}\ {\mathrm{of}\ \mathrm{Stenosis}}_{\mathrm{pre}-\mathrm{angioplasty}}\right]\ \mathrm{x}\ 100 $$

## Hemodynamic evaluation using parametric colour-coded DSA

### Difference in time to peak contrast density (dTTP) (Fig. 2)

Pre- and post-PTA sequences were post-processed and converted into colour images. For each image, one region of interest (ROI) was chosen pre- and post-stenosis. Time to peak (TTP) contrast enhancement or time at which each pixel reached its peak intensity was computed for each ROI. The difference in time to peak (dTTP) contrast density between these 2 ROIs pre- and post-PTA provides information on hemodynamic changes and were compared before and after intervention.

**Fig. 2:**
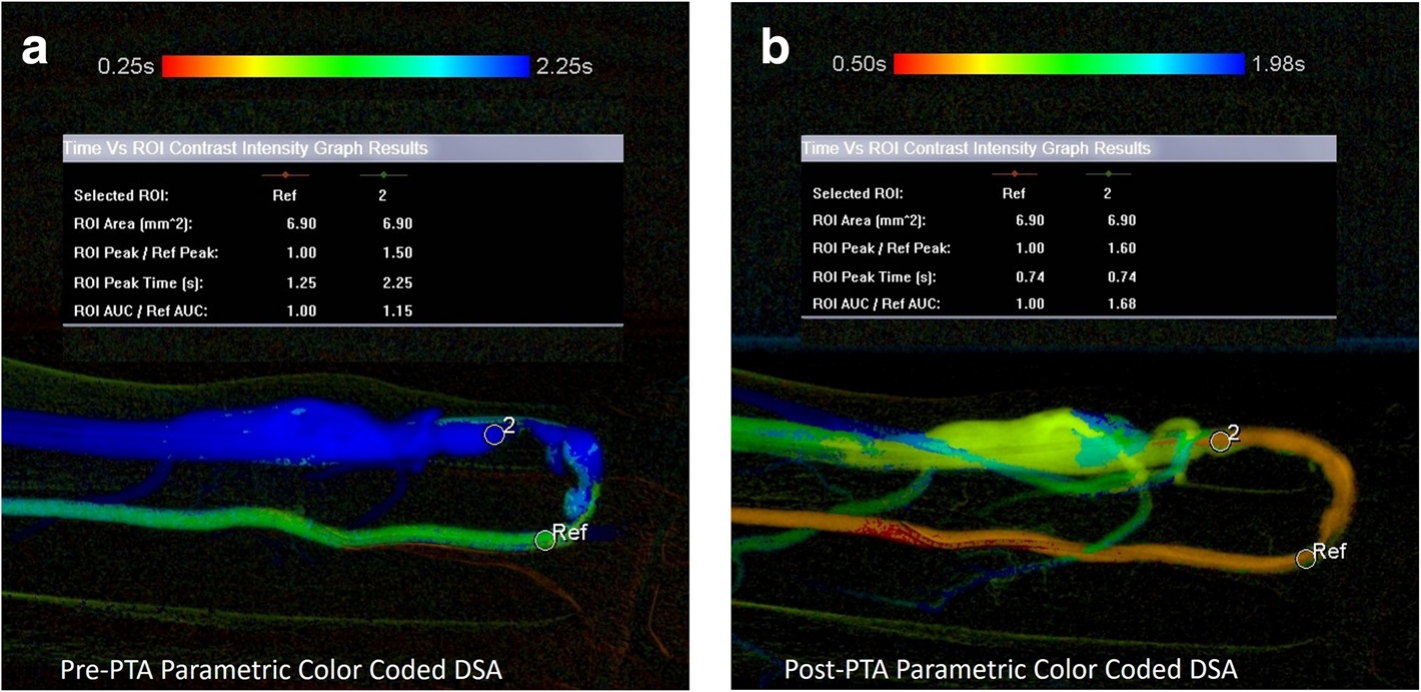
Pre- and post-PTA parametric colour-coded DSA with region of interests (ROI) of the same AVF shown in Fig. 6. DSA sequences were post-processed and parametric colour-coded DSA was generated. ROIs were chosen pre- and post-stenosis. The differences of TTP (dTTP) values between these 2 ROIs pre- and post-PTA were generated. (**a**) Pre-PTA dTTP was 1 s. (**b**) Post-PTA dTTP was 0, indicating contrast flow was faster after intervention

### Quantification of contrast wash out (Fig. 3)

Time attenuation curves (TAC) pre- and post-treatment were generated using opacity data per ROI per time point calculated by the software. 50% contrast wash out time or time taken for 50% attenuation in contrast density after the peak intensity was obtained and compared pre- and post-PTA.

**Fig. 3:**
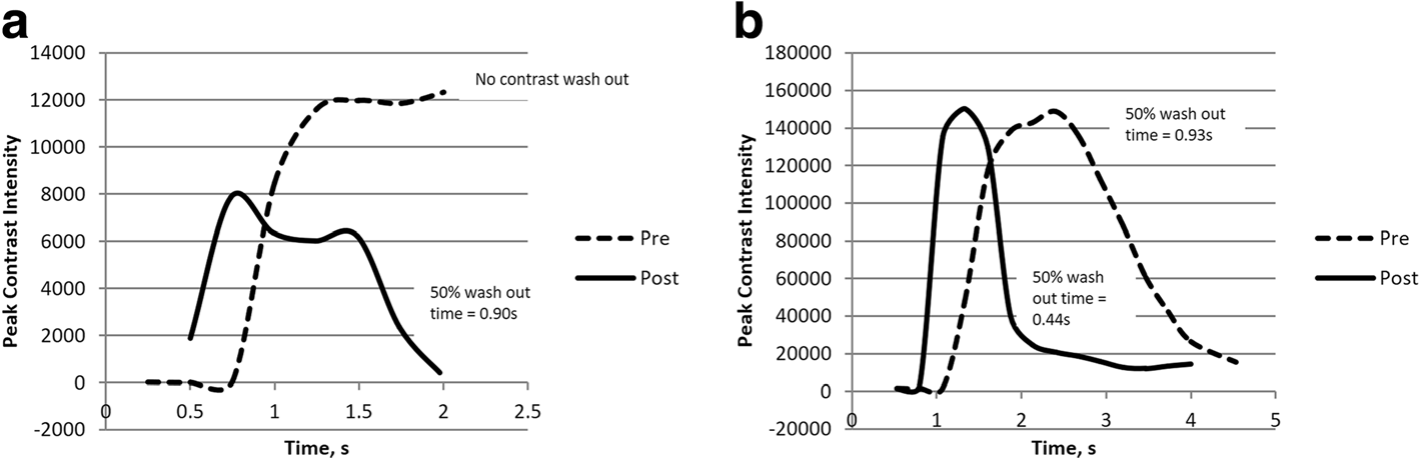
Quantification of contrast wash out time using time attenuation curve (TAC). (**a**) Time vs. contrast intensity curve of the AVF shown in Fig. 6. Pre-PTA TAC showed no contrast washout during the entire DSA captured, indicating slow flow. Post-PTA TAC exhibited contrast wash out over time and the time taken for 50% contrast wash out was 0.9 s. (**b**) Time vs. contrast intensity curve of an AVG treatment showing contrast wash out over time in both pre- and post-PTA TAC. The time taken for 50% contrast wash improved from 0.93 to 0.44 s

#### Statistical analysis

Data was presented as frequency (percentage) for categorical variable; mean (standard deviation) for normally distributed or median (interquartile range (IQR)) for non-normally distributed continuous variables. Percent of stenosis, dTTP and 50% contrast washout time were compared using paired sample t-test or Wilcoxon signed-rank test depending on data type. Correlations between pre-PTA percent of stenosis vs. dTTP and post-PTA change in percent of stenosis vs. change in dTTP were obtained using Spearman’s rank correlation test. Data analyses were performed using SPSS Statistics for Windows, Version 21 (Armonk, NY:IBM Corp).

## Results

The baseline demographics and characteristics of the patients is summarized in Table 1. The study subjects have a mean age of 66.6 ± 9.3 years. AVF was the predominant vascular access (75%) with median access age of 3.1 (IQR 0.7, 5.3) years. Two third of the lesions studied were outflow stenosis (60%).

**Table 1:** Baseline Demographics of Patients

Demographics	Mean ± standard deviation or Median (IQR) or n (%)
Age, years	66.6 ± 9.3
Female, n (%)	12 (60)
Age of Access, years	3.1 (0.7, 5.3)
Reason for Intervention, n (%)	
Decreased access flow	7 (35)
High venous pressure	5 (25)
Weak thrill	3 (15)
Arm swelling	4(20)
Thrombosis	1 (5)
Access Type, n (%)	
Arteriovenous fistula (AVF)	15 (75)
Radiocephalic	4 (20)
Brachiocephalic	8 (40)
Brachiobasillic	3 (15)
Arteriovenous graft (AVG)	5 (25)
Brachiobasillic	2 (10)
Femoral-artery-vein	3 (15)
Number of lesions, n (%)	
1	16 (80)
2	4 (20)

Information on each vascular access, location of lesions, anatomic and hemodynamic parameters obtained, and patency is summarized on Table 2. All inflow lesions were located at the juxta-anastomotic segment. Conversely, outflow lesions were located at various anatomical sites including peripheral vein, cephalic arch, graft vein junctions and central veins. (Table 2) All cases demonstrated variable anatomical improvements following angioplasty. Improvements in dTTP were observed in 15 patients. Lack of improvement in dTTP was noted in 5 patients. Of which, 3 of the patients have inflow lesions with less than 50% stenosis. The remaining 2 patients had outflow stenoses with more than 50% anatomical improvement. Pre-PTA TAC of 8 out of 20 patients did not show attenuation in contrast density due to high resistance in contrast flow caused by severe stenosis. Contrast washout was exhibited in all patients post-PTA. (Fig. 3) Comparison of 50% contrast washout time pre- and post-PTA was only possible for 12 patients. Of which, 10 patients exhibited improvement in 50% contrast washout time while 2 patients did not. All patients underwent successful haemodialysis through the index vascular access with blood flow of at least 200mls/min after intervention. 18 patients have recurrent stenosis requiring repeat interventions at the time of patency measurement. The median primary patency was 3.9 (IQR 2.5, 10.3) months. (Table 2).

**Table 2:** Characteristics of Vascular Access, Anatomical and Hemodynamic Parameters

Cases	Access	Anastomosis	Number of Stenosis	Anatomical site assessed	Anatomical Parameters, %		Hemodynamic Parameters, s			Time to Next PTA, months
					% of stenosis	Change in % of Stenosis	dTTP	Improvement in dTTP	Improvement in 50% Contrast Wash Out Time	
1	AVF	Brachio-cephalic	1	Cephalic arch	74.76	50.15	0.25	0.80	0.97	Nil^c^
2	AVF	Brachio-cephalic	2^a^	Juxta-anastomosis	20.69	33.55	0.26	−0.01	0.14	11.27
3	AVF	Radio-cephalic	1	Juxta- anastomosis	50.86	66.33	0.80	0.01	0.61	5.29
4	AVF	Radio-cephalic	1	Juxta- anastomosis	68.89	92.20	0.51	0.80	No wash out	3.91
5	AVF	Brachio-cephalic	2^a^	Juxta- anastomosis	44.10	56.30	3.72	0.25	0.38	2.46
6	AVF	Radio-cephalic	1	Juxta- anastomosis	45.47	92.78	0.25	3.47	No wash out	Nil^c^
7	AVF	Brachio-cephalic	2^a^	Juxta- anastomosis	42.04	19.07	0.26	0	−0.14	15.87
8	AVF	Brachio-cephalic	1	Juxta- anastomosis	30.96	33.82	1.00	−0.01	0.34	11.96
9	AVF	Brachio-basillic	1	Draining vein	72.37	83.06	0.80	0.53	No wash out	1.64
10	AVF	Radio-cephalic	1	Juxta- anastomosis	75.93	60.41	0.53	1.00	No wash out	7.33
11	AVG	Brachio-basillic	1	Graft vein junction	74.57	95.93	0.53	0.27	0.49	0.59
12	AVG	Brachio-basillic	2^b^	Draining vein	83.63	54.42	1.06	0.81	No wash out	3.19
13	AVG	Femoral artery-vein	1	Graft vein junction	56.56	71.63	0.26	0	No wash out	3.58
14	AVG	Femoral artery-vein	1	Graft vein junction	52.86	86.59	0.54	0.28	0.94	4.63
15	AVG	Femoral artery-vein	1	Graft vein junction	60.12	81.49	0.25	0.25	No wash out	3.91
16	AVF	Brachio-basillic	1	Brachiocephalic vein	100.00	50.08	0.54	0.54	0.12	2.46
17	AVF	Brachio-cephalic	1	Brachiocephalic vein	91.47	32.59	0.27	0.01	No wash out	2.76
18	AVF	Brachio-cephalic	1	Subclavian vein	77.98	41.22	0.80	0.51	0.06	2.07
19	AVF	Brachio-cephalic	1	Subclavian vein	89.33	51.25	0.27	0	−0.37	1.91
20	AVF	Brachio-basillic	1	Brachiocephalic vein	84.34	69.86	0.26	0.26	0.12	7.56

^a^Proximal lesions were treated before distal lesions, therefore anatomical and hemodynamic parameters of the distal lesions were obtained for comparison

^b^Both lesions were treated simultaneously

^c^Intervention free at last follow-up

### Anatomical and hemodynamic assessments (Table 3)

Mean percent of stenosis improved from 64.85 ± 21.39 to 24.73 ± 17.03% after PTA (*p* < 0.0001). Median dTTP improved from 0.52 (IQR 0.26, 0.8) to 0.25 (IQR 0, 0.26) seconds (*p* = 0.001). Median 50% contrast washout time also improved from 0.77 (IQR 0.39, 1.17) to 0.42 (IQR 0.23, 0.59) seconds (*p* = 0.031).

**Table 3:** Pre- and Post-percutaneous Angioplasty Comparison of Anatomic and Hemodynamic Parameters

Parameters, *n* = 20	Mean ± standard deviation or Median (IQR)	*P* – value
Percent of Stenosis, %		< 0.0001
Pre-PTA	64.85 ± 21.39	
Post-PTA	24.73 ± 17.03	
dTTP, s		0.001
Pre-PTA	0.52 (0.26, 0.8)	
Post-PTA	0.25 (0, 0.26)	
50% Contrast Washout Time, s		0.031
Pre-PTA	0.77 (0.39, 1.17)	
Post-PTA	0.42 (0.23, 0.59)	

### Scatter plot diagrams and correlation analysis

**Fig. 4:**
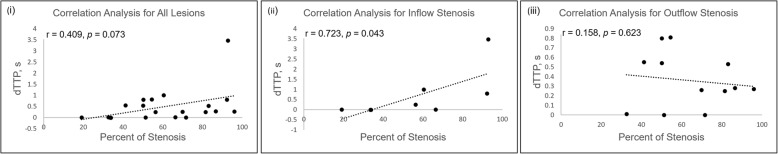
Correlation between Pre-PTA dTTP and Percent of Stenosis. Scatter plot diagrams of dTTP vs. percent of stenosis for (**i**) all accesses, (**ii**) accesses with inflow stenosis, (**iii**) accesses with outflow stenosis

**Fig. 5:**
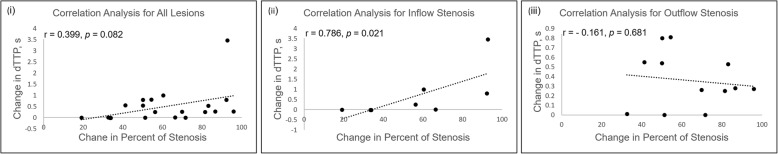
Correlation between Post-PTA Change in dTTP and Change in Percent of Stenosis. Scatter plot diagrams of post-PTA change in dTTP vs. change in percent of stenosis for (**i**) all accesses, (**ii**) accesses with inflow stenosis, (**iii**) accesses with outflow stenosis

Scatter plot diagrams of dTTP vs. percent of stenosis (Fig. 4) and change in dTTP vs. change in percent of stenosis (Fig. 5) were obtained. Correlation analysis revealed no significant correlation for pre-PTA dTTP vs. percent of stenosis (*r* = 0.409, *p* = 0.073) and post-PTA change in dTTP vs. change in percent of stenosis (*r* = 0.399, *p* = 0.082). However, when the lesions were subdivided into inflow and outflow lesions, significant correlation was seen for dTTP vs. percent of stenosis for inflow stenosis (*n* = 8, *r* = 0.723, *p* = 0.043) but not for outflow stenosis (*n* = 12, *r* = 0.158, *p* = 0.623). Similarly, there was significant correlation for post-PTA change in dTTP vs. percent change in stenosis for inflow lesions (*r* = 0.786, *p* = 0.021) but not for outflow lesions (*r* = − 0.161, *p* = 0.681).

**Fig. 6:**
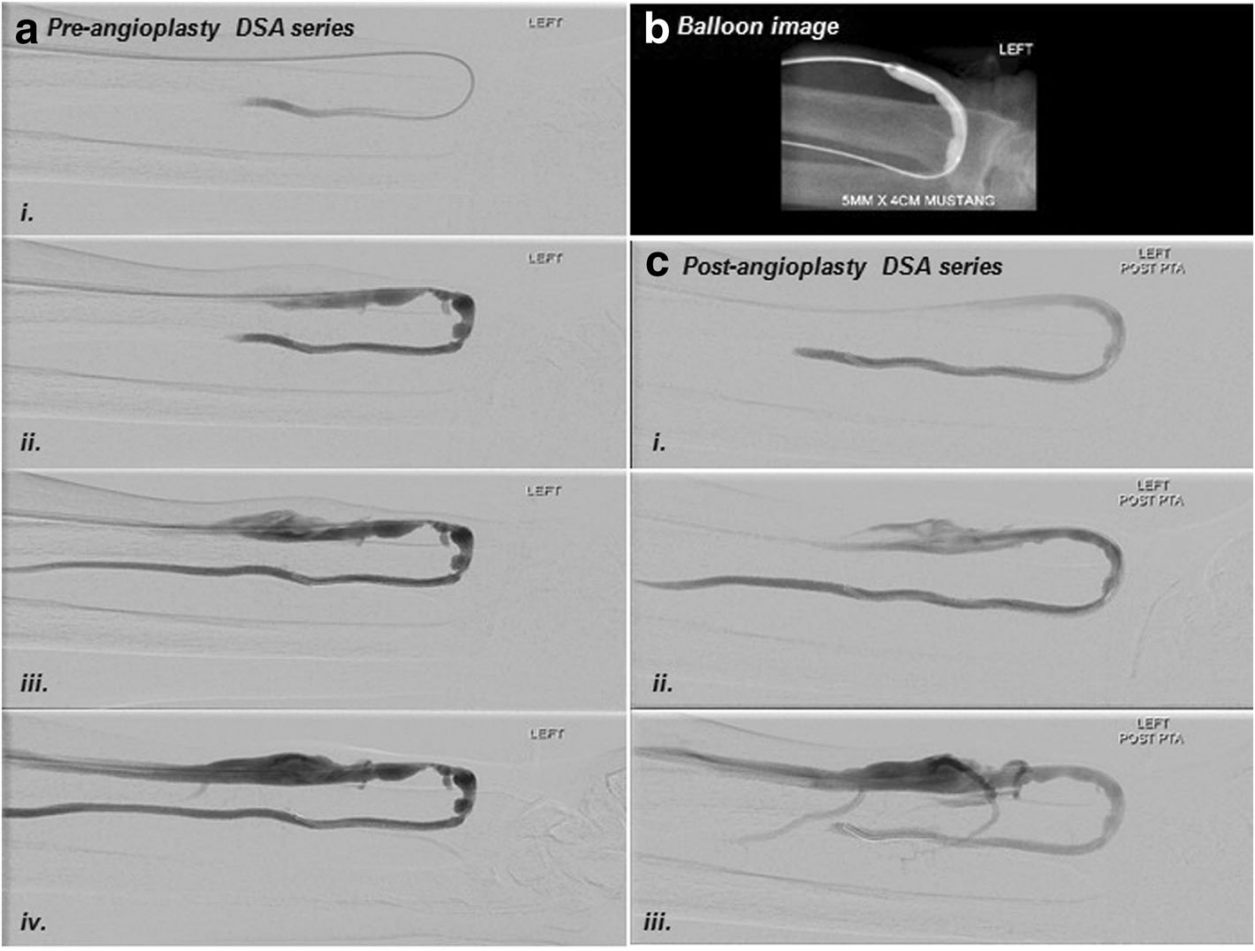
Images of a left radio-cephalic arterio-venous fistula (AVF) with stenosis along juxta-anastomosis segment. (**i**): Frame by frame black and white images showing juxta-anastomotic segment stenosis pre-PTA. (**ii**): An angioplasty balloon was used to treat the stenosis, complete effacement of the balloon was seen. (**iii**): Frame by frame black and white images showing interval improvement of the juxta-anastomotic segment stenosis with more prominent opacification of collaterals from outflow vein after PTA

## Discussions

In this study, we have explored the use of parametric colour coded DSA in the management of dysfunctional dialysis access. The hallmark of successful interventions in dialysis access has always been successful effacement of stenosis (Fig. 6). However, the functional aspect is equally important, and we hypothesize that parametric colour-coded DSA will provide a quantitative assessment of interventional outcome. We demonstrated significant improvements in dTTP and time taken for 50% contrast attenuation post-PTA as surrogates for improvement in access flow after PTA and significant correlations of anatomical vs. hemodynamic assessments pre- and post PTA. This information might be potentially useful for assessing the significance of lesion and adequacy of treatment during percutaneous intervention of haemodialysis vascular access.

Maintaining sufficient access flow is crucial for adequate hemodialysis [13]. Secondary patency of vascular access after PTA however remains dismal, with re-stenosis and re-intervention rates reported to be up to 60% at 6 months [14, 15]. Visual analysis of conventional two-dimensional DSA sequences typically requires viewing a series images and evaluating the differences in images which the contrast medium creates as it passes through the obstructions in the vessels. Browsing through the entire black and white filling sequences frame by frame during the procedure can be time consuming and is subjected to interpersonal variability. Lack of correlation between angiographic visual assessment and hemodynamic performance of vascular access has been demonstrated in many previous studies [16, 17]. Hence, risk of poor long-term outcome despite successful intervention if treatment adequacy is relied solely on angiographic visual assessment.

Several techniques for assessment of vascular stenosis and intra-procedural real-time hemodynamic assessments in hemodialysis accesses have been described. Magnetic resonance angiography (MRA) and computed tomography angiography (CTA) have been reported to provide high quality diagnostic images for vascular stenosis [3, 18–21]. Time resolved MRA could be a useful screening tool as it is non-invasive and is able to detect vascular stenosis with smaller dose of contrast [20, 21]. Catheter based thermodilution on the other hand is a method to extrapolate intra-access blood flow from thermodilution equation and the thermal properties of blood and saline [17, 22]. It was reported to correlate well with the current gold standard of ultrasound dilution method but the major drawbacks were high cost and high technical failure rates as correct placement of catheter may not always be possible [22]. Ultrasound Doppler flow measurement is another method which is readily available in most interventional radiology suite. However, it is operator dependent and its precision is limited by uncertainties in the measure of vessel diameter, Doppler angle and blood velocity determination. Utility of intra-access static pressure was also investigated and found to be a simple and potentially useful hemodynamic indicator during PTA. An elevated static pressure which declined to normal represented adequately treated outflow stenosis while restoration of a negative static pressure PTA to normal reading could represent adequately treated inflow stenosis [23]. Unfortunately, static pressure may be normal when there is co-existing inflow and outflow stenosis exerting opposing hemodynamic effects of equal magnitude, making hemodynamic assessment impossible as there will not be a change in static pressure after treatment [23].

Parametric colour-coded DSA provides an alternative method for hemodynamic assessment. It is easy to use, non-invasive and does not involve use of additional expensive equipment. More importantly, hemodynamic parameters can be instantly generated during the procedure to enable real-time therapeutic planning. Vascular access with multifocal stenosis is not uncommonly encountered during interventional procedures. The approach of treating all encountered lesions has not been shown to improve access patency [24]. Determining whether a visible lesion is clinically indicated for treatment may be challenging on table. Hemodynamic parameters from colour-coded DSA could therefore be a useful adjunctive tool to help determine the necessity and adequacy of treatment of every lesion seen on venography, resulting in potential cost saving and reduction in radiation exposure.

Although pre-PTA 50% contrast washout times were not available in 8 cases, the TACs of these cases still provided some insight on the hemodynamic parameters of the accesses. In these cases, stenosis was likely hemodynamically significant as resistant to contrast flow has resulted in slow contrast attenuation below 50% during the entire DSA sequences captured (Fig. 3). The delay in contrast attenuation was also congruent with anatomical assessment as 7 out of 8 of these patients have lesions with more than 50% stenosis.

Significant correlations were noted between anatomic and hemodynamic parameters pre- and post-PTA for inflow stenosis but not outflow stenosis. We postulated that this could be due to the different vascular characteristics of the different outflow components. For example, size, elasticity and curvature are different in central veins compared to peripheral veins [25]. Furthermore, when the access has more than one outflow such as in a radiocephalic fistula, the quantity, size and location of the outflow veins may also result in inaccuracy during hemodynamic assessment.

Although the effect of colour was not evaluated in our study, previous studies on colour-coded DSA have suggested that colour coding provided images of high quality that were not impaired by motion artefact [10]. Furthermore, colour is known to improve visual search and identification performance [26]. The display of colour can be used to enhance human perception of medical information and has been successfully used in computed tomography, magnetic resonance imaging and sonograms. As human eyes perceive colour differentiation over a wide range, changes in signal intensity can be easily distinguishable with the naked eye [27]. Evaluation of vascular access with parametric colour coding could therefore be easier and more accurate than conventional black and white DSA [9].

We acknowledge that there were several limitations of this study. The major limitation of our study was the small sample size from a single centre which limits its power and generalizability. Furthermore, due to the retrospective nature of this study where hand injection of contrast during DSA acquisition was the standard of care, differences in pressure during injection, volume of contrast used before and after PTA may affect the hemodynamic parameters measured. However, contrast injections for all the cases included in the study were by the same operator throughout the procedure. Our method of retrospective case selection may also introduce selection bias. Although all PTA were deemed successful clinically and radiologically, we did not examine the effect of hemodynamic parameters on resolution of clinical symptoms nor correlate the results with direct flow measurement or indirect pressure measurement. Estimation of percentage of luminal stenosis using diameter measurement is not the most ideal as this calculation is a one-dimensional view. Vessels are circular or elliptical, and the stenoses are frequently asymmetric when viewed in cross section. Estimating the luminal stenosis using one plane in this study may therefore result in inaccuracy. Additionally, although there was statistical improvement in the TTP and contrast wash out time post angioplasty, we are unable to determine the cut off values to define success or adequacy of angioplasty due to the small sample size. Nevertheless, within the limits of the study, we have demonstrated that parametric colour-coded DSA may be able to provide potentially useful quantitative information on hemodynamic changes in vascular access during PTA, especially for inflow lesions. This is relevant to clinical practice as the aim of PTA is to restore sufficient flow within the dialysis access for hemodialysis.

## Conclusions

This pilot study suggests that adjunctive use of parametric colour-coded DSA may have positive impact in haemodialysis vascular access interventions. We proposed that a larger prospective study using standardized contrast injection rate with correlation of hemodynamic parameters with the gold standard ultrasound dilution technique of intra-access flow should be performed to further evaluate the effectiveness and reliability of colour-coded DSA in haemodynamic assessment of haemodialysis vascular access.

## Abbreviations

AVF: Arteriovenous fistula
AVG: Arteriovenous graft
DSA: Digital subtraction angiography
dTTP: Difference in time to peak
ESRF: End stage renal failure
PTA: Percutaneous transluminal angioplasty
ROI: Regions of interest
TAC: Time attenuation curve

## References

[1] Schinstock CA, Albright RC, Williams AW, Dillon JJ, Bergstralh EJ, Jenson BM (2011). Outcomes of arteriovenous fistula creation after the fistula first initiative. Clin J Am Soc Nephrol.

[2] Roy-Chaudhury P, Sukhatme VP, Cheung AK (2006). Hemodialysis vascular access dysfunction: a cellular and molecular viewpoint. J Am Soc Nephrol.

[3] Razek AA, Saad E, Soliman N, Elatta HA (2010). Assessment of vascular disorders of the upper extremity with contrast-enhanced magnetic resonance angiography: pictorial review. Jpn J Radiol.

[4] NKF-DOQI. Clinical Practice Guidelines for Vascular Access. Am J Kidney Dis. 2006;48:S176–247. 10.1053/j.ajkd.2006.04.029 16813989

[5] Mishler R, Sands JJ, Ofsthun NJ, Teng M, Schon D, Lazarus JM (2006). Dedicated outpatient vascular access center decreases hospitalization and missed outpatient dialysis treatments. Kidney Int.

[6] Doelman C, Duijm LEM, Liem YS, Froger CL, Tielbeek AV, Donkers-van Rossum AB (2005). Stenosis detection in failing hemodialysis access fistulas and grafts: comparison of color Doppler ultrasonography, contrast-enhanced magnetic resonance angiography, and digital subtraction angiography. J Vasc Surg.

[7] Leontiev O, Mondschein JI, Dagli MS, Clark TWI, Soulen MC, Stavropoulos SW (2013). Catheter-based Intraaccess blood flow measurement as a problem-solving tool in hemodialysis access intervention. J Vasc Interv Radiol.

[8] Gray RJ, Sacks D, Martin LG, Trerotola SO (2003). Reporting standards for percutaneous interventions in dialysis access. J Vasc Interv Radiol.

[9] Strother CM, Bender F, Deuerling-Zheng Y, Royalty K, Pulfer KA, Baumgart J (2010). Parametric color coding of digital subtraction angiography. AJNR Am J Neuroradiol.

[10] Golitz P, Struffert T, Lucking H, Rosch J, Knossalla F, Ganslandt O (2013). Parametric color coding of digital subtraction angiography in the evaluation of carotid cavernous fistulas. Clin Neuroradiol.

[11] Lin CJ, Hung SC, Guo WY, Chang FC, Luo CB, Beilner J (2012). Monitoring peri-therapeutic cerebral circulation time: a feasibility study using color-coded quantitative DSA in patients with steno-occlusive arterial disease. AJNR Am J Neuroradiol.

[12] Su H, Lou W, Gu J (2015). Clinical values of hemodynamics assessment by parametric color coding of digital subtraction angiography before and after endovascular therapy for critical limb ischaemia. Zhonghua Yi Xue Za Zhi.

[13] Whittier WL (2009). Surveillance of hemodialysis vascular access. Semin Interv Radiol.

[14] Kim WS, Pyun WB, Kang BC (2011). The primary patency of percutaneous Transluminal angioplasty in hemodialysis patients with vascular access failure. Korean Circ J.

[15] Agarwal SK, Nadkarni GN, Yacoub R, Patel AA, Jenkins JS, Collins TJ (2015). Comparison of cutting balloon angioplasty and percutaneous balloon angioplasty of Arteriovenous fistula stenosis: a meta-analysis and systematic review of randomized clinical trials. J Interv Cardiol.

[16] Ahya SN, Windus DW, Vesely TM (2001). Flow in hemodialysis grafts after angioplasty: do radiologic criteria predict success?. Kidney Int.

[17] Goyal A, Orth RC, Parekh RS, Wolfson T, Gamst A, Kuo MD (2011). Endpoints for hemodialysis access procedures: correlation between fistulography and intraaccess blood flow measurements. J Vasc Interv Radiol.

[18] Abdel Razek AAK, Denewer AT, Hegazy MAF, Hafez MTA (2014). Role of computed tomography angiography in the diagnosis of vascular stenosis in head and neck microvascular free flap reconstruction. Int J Oral Maxillofac Surg.

[19] Lin YP, Wu MH, Ng YY, Lee RC, Liou JK, Yang WC (1998). Spiral computed tomographic angiography--a new technique for evaluation of vascular access in hemodialysis patients. Am J Nephrol.

[20] Razek AA, Gaballa G, Megahed AS, Elmogy E (2013). Time resolved imaging of contrast kinetics (TRICKS) MR angiography of arteriovenous malformations of head and neck. Eur J Radiol.

[21] Zhang J, Hecht EM, Maldonado T, Lee VS (2006). Time-resolved 3D MR angiography with parallel imaging for evaluation of hemodialysis fistulas and grafts: initial experience. AJR Am J Roentgenol.

[22] Heerwagen ST, Hansen MA, Schroeder TV, Ladefoged SD, Lonn L (2012). Blood flow measurements during hemodialysis vascular access interventions--catheter-based thermodilution or Doppler ultrasound?. J Vasc Access.

[23] Asif A, Besarab A, Gadalean F, Merrill D, Rismeyer AE, Contreras G (2006). Utility of static pressure ratio recording during angioplasty of arteriovenous graft stenosis. Semin Dial.

[24] Leontiev O, Shlansky-Goldberg RD, Stavropoulos SW, Mondschein JI, Itkin M, Clark TWI (2014). Should all inflow Stenoses be treated in failing autogenous hemodialysis fistulae?. J Vasc Interv Radiol.

[25] Agarwal AK (2015). Endovascular interventions for central vein stenosis. Kidney Res Clin Pract.

[26] Cole BL, Maddocks JD, Sharpe K (2004). Visual search and the conspicuity of coloured targets for colour vision normal and colour vision deficient observers. Clin Exp Optom.

[27] Benndorf G (2010). Color-coded digital subtraction angiography: the end of a monochromatic era?. AJNR Am J Neuroradiol.

